# Strategy to select nasopharyngeal cancer patients for adaptive radiotherapy

**DOI:** 10.3389/fonc.2025.1653060

**Published:** 2025-09-29

**Authors:** Heleen Bollen, Elena Onorati, Annouschka Laenen, Anneleen Goedgebeur, Sandra Nuyts

**Affiliations:** ^1^ Laboratory of Experimental Radiotherapy, Department of Oncology, University of Leuven, Leuven, Belgium; ^2^ Department of Radiation Oncology, Leuven Cancer Institute, University Hospitals Leuven, Leuven, Belgium; ^3^ Department of Radiation Oncology, Università Campus Bio-Medico di Roma, Rome, Italy; ^4^ Leuven Biostatistics and Statistical Bioinformatics Center, University of Leuven, Leuven, Belgium

**Keywords:** adaptive radiotherapy (ART), nasopharyngeal carcinoma, proton therapy, predictors, IMRT (intensity modulated radiation therapy)

## Abstract

**Background:**

Nasopharyngeal cancer (NPC) patients experience significant anatomical changes during radiation treatment (RT). Adaptive radiotherapy (ART) can be initiated in response to specific events or at scheduled intervals during treatment, prompting questions about patient selection, timing, and reliable triggers for re-CT and replanning. This study aimed to develop a method for selecting NPC patients for ART before treatment and identify the optimal timing for its implementation.

**Materials and methods:**

We retrospectively evaluated NPC patients treated at University Hospitals Leuven between 2016 and 2023, assessing volumetric changes in the parotid glands, air cavities, maxillary sinuses, and body contour at nasopharyngeal and low-neck levels throughout treatment. Structures were contoured on the initial simulation CT, weekly CBCTs, and final treatment day, resulting in eight measurements per parameter. Body contour changes were evaluated at the nasopharyngeal level (odontoid process) and neck level (lower edge of the third cervical vertebra). Measurements included volume, transverse diameters, and radius angles of 50° and 310°. Kaplan-Meier analysis was used for overall survival (OS) and local control (LC), while longitudinal volumetric data were analyzed with linear mixed models. Continuous variables were dichotomized to create a binary variable, for the purpose to define cut-off values for significant predictive variables.

**Results:**

Of the 47 NPC patients analyzed, 2- and 5-year loco-regional control rates were 95%, with overall survival rates of 100% and 93%, respectively. Significant reductions in parotid gland volume and body contour were observed, particularly between weeks 3 and 4. Predictors of parotid gland volume reduction included bulky nodal disease and larger initial parotid volume, with thresholds of 15 mm and 56 cc, respectively. Body contour changes during the fourth week correlated with high N-stage (≥ N3), higher initial BMI (>28 kg/m²), bulky lymph nodes (15 mm), higher initial mean planned dose to the ipsilateral parotid gland (> 31 Gy) and larger initial primary tumor Clinical Target Volume (> 93 cc). Induction chemotherapy was significantly associated with fewer sinonasal air cavity changes than concomitant chemoradiotherapy.

**Conclusion:**

It is recommended to re-evaluate the RT plan during the period between fractions 15 and 20 of treatment. Patients treated for NCP could be selected for ART based on the following clinical criteria at diagnosis: N3 or higher classification, presence of a bulky lymph node larger than 15 millimeters, initial BMI exceeding 28 kg/m², mean planned dose to the ipsilateral parotid glands greater than 31 Gy, cumulative volume of the parotid glands greater than 56 cc, high-dose CTV of the primary tumor greater than 93 cc, and receiving RT with no prior induction chemotherapy. Validation of these pre-treatment clinical predictors in a large, prospective dataset is essential before clinical usage.

## Introduction

Nasopharyngeal carcinoma (NPC) is a unique subtype of head and neck cancer (HNC), characterized by distinct etiology and epidemiological patterns (1). Given its anatomical location and intrinsic radiosensitivity, radiotherapy (RT) presents the primary treatment modality for NPC, often in combination with chemotherapy, depending on the disease stage (2). However, treating NPC with radiation presents significant challenges due to its proximity to critical structures. While RT is effective, it carries the risk of both acute and long-term side effects, including feeding tube dependence, hearing impairment, temporal lobe necrosis, and cognitive decline (3–7). Given the generally good prognosis for NPC patients following RT, minimizing these long-term adverse effects is crucial. Proton therapy (PT) has emerged as a promising alternative due to its superior dose distribution and its potential to reduce acute toxicities compared to IMRT (8). Yet, PT also presents unique challenges, such as its sensitivity to changes in tissue density due to the uncertainty of the exact location of the Bragg peak and the sharp distal dose fall-off of protons. Factors like positioning errors, artifacts, tissue deformations, and anatomical changes —often caused by weight loss or tumor response in HNC patients —can shift the Bragg peak (9).

Adaptive radiotherapy (ART) offers a dynamic solution to the variability in patient anatomy during treatment. By incorporating imaging throughout the course of therapy, ART enables adjustments to the treatment plan, enhancing tumor coverage while minimizing dose to OARs (10). However, the optimal timing and criteria for plan adaptation remain unclear, particularly in the context of PT (11). This study aims to address these uncertainties by objectifying anatomical changes occurring during treatment, identifying pre-treatment clinical predictors for these changes, and determining the time points at which the most significant alterations are observed. The ultimate goal is to refine ART strategies, improving the therapeutic ratio in NPC treatment.

## Materials and methods

### Patient selection and data collection

This retrospective study included patients with histologically confirmed NPC, treated with RT at UH Leuven between 2016 and 2023. Each patient underwent a thorough clinical and radiological evaluation, including MRI of the head and neck and a total body FDG PET-CT scan. Clinical staging was performed according to the American Joint Committee on Cancer (AJCC) 8th edition. After discussion by a multidisciplinary institutional tumor board, patients received treatment at UH Leuven, consisting of RT alone or in combination with chemotherapy, based on disease stage and associated risk factors. Data on patient characteristics, treatment modalities, plan specifications, and anatomical changes during treatment were collected retrospectively. Follow-up adhered to ESMO-EURACAN clinical practice guidelines for NPC (2), with imaging (MRI or FDG PET/CT) three months after RT, then every six months up to the 3rd year, and endoscopic assessment every 3 months in the first year, every 6 months in the second and third years and annually thereafter. Overall survival (OS) was measured from RT initiation to death from any cause. Disease metastasis (DM) was tracked from the start of RT to distant recurrence. Locoregional control (LRC) was assessed from RT initiation to recurrence. Patients still under follow-up or lost to follow-up were censored at their last known date of survival (for OS) or recurrence-free status (for LRC and DM). This study was approved by the Ethics Committee of the University Hospitals of Leuven (S62953).

### Radiotherapy

Patients were positioned supine and immobilized using thermoplastic masks covering the head and shoulders. Target volumes and organs at risk were contoured by the radiation oncologist following international guidelines (2). Gross Tumor Volume (GTV) included all detectable disease from CT, MRI, clinical data, and endoscopy. Lymph nodes greater than 1 cm or with necrotic centers were included in the GTV. High-risk Clinical Target Volume (CTV 70) encompassed the primary disease site and grossly involved lymph nodes, with a 1 cm margin added to the GTV. Intermediate-risk CTV (CTV 59.4-P and CTV 59.4-N) was defined by adding a 5 mm margin to the CTV 70-P and CTV 70-N, respectively. CTV 59.4-P also included the entire nasopharynx, clivus, skull base, pterygoid fossae, parapharyngeal space, sphenoid sinus, and portions of the nasal cavity and maxillary sinuses. CTV 59.4-N included the retropharyngeal nodes and elective neck levels Ib to V bilaterally. Low-Risk CTV (CTV 54) covered the lower neck lymph nodes in cases without gross lymph node involvement. For photon therapy, Planning Target Volumes (PTV 70, PTV 59.4, PTV 54) were generated by adding a 5 mm margin to the respective CTVs. PT plans accounted for setup uncertainties of 3 mm above the mandible and 5 mm below. Treatment plans were developed by medical physicists using Eclipse for photon plans and RayStation for proton plans, adhering to international dose prioritization and acceptance criteria. Dose specifications included: PTV 70: 69.96 Gy in 33 fractions (EQD2 70.66 Gy), PTV 59.4: 59.4 Gy in 33 fractions (EQD2 58.41 Gy) and PTV 54: 54 Gy in 33 fractions (EQD2 52.38 Gy). Photon treatments were delivered using VMAT with Simultaneous Integrated Boost (SIB), while proton treatments used Intensity Modulated Proton Therapy (IMPT) with robust optimization. Daily Cone-Beam CTs (CBCT) were obtained for accurate patient positioning. In-silico proton plans were created for randomly selected patients to compare organ-at-risk dose delivery with equivalent target coverage.

### Chemotherapy

Patients received concurrent chemotherapy with high dose cisplatin. Induction chemotherapy included cisplatin alone or in combination with fluorouracil and docetaxel (TPF), gemcitabine, or taxanes.

### Delineation of anatomical changes

The study focused on changes in the parotid glands, air cavities, maxillary sinuses, and body contour at the nasopharyngeal and low-neck levels. Each structure was contoured on the initial simulation CT, each weekly CBCT, and on the last day of treatment, yielding eight measurements per parameter. Volume changes in the parotid glands were measured by delineating the parotid glands weekly and calculating the mean volume of both parotid glands (**Supplementary Materials 1A**). Air cavities at the nasopharyngeal level were measured as a way to evaluate tumor regression indirectly (**Supplementary Materials 2A**). Maxillary sinus volumes were measured to assess potential non-tumor-related variations impacting proton planning (**Supplementary Materials 3A**). Body contour changes were assessed at two levels: 1) nasopharyngeal level: volume, measured in cc, at the level of the odontoid process (**Supplementary Materials 4A**); 2) neck level: volume, measured in cc, at the lower edge of the third cervical vertebra (**Supplementary Materials 5A**). Additionally, for both the nasopharyngeal and neck regions, radius angles of 50° and 310° were established and measured in centimeters. These measurements were taken from the isocenter of the CT/CBCT to the skin surface at 50° and 310° angles, respectively, corresponding to the levels of the odontoid process and the third cervical vertebra (C3) (**Supplementary Materials 6A, 7A**). These specific angles were selected because they represent the typical entry trajectory of the anterior proton beam in our planning protocol, where anatomical changes along this path are expected to have the greatest influence on PT dose distribution. Half-fan CBCT images with iterative reconstruction were obtained using Halcyon linacs, in line with University Hospitals Leuven’s standard practice for Head and Neck Image-Guided Radiotherapy (IGRT). In instances where the Halcyon machine required maintenance, patients were occasionally treated on a Truebeam linac, following a comparable half-fan CBCT onboard imaging IGRT protocol.

### Statistical analysis

Overall survival (OS) was defined as the time from diagnosis to death or the last follow-up, and local control (LC) as the time from diagnosis to local recurrence/progression or last follow-up. OS and LC were estimated using the Kaplan-Meier method. Linear mixed models were used for the estimation of longitudinal volumetric evolutions, and for the analysis of predictive effects of clinical and dosimetric characteristics on volumetric changes. In a second phase, an exploratory analysis was conducted to identify the specific thresholds that most effectively distinguish patients experiencing pronounced anatomical changes during RT from those who did not. To establish binary variables and determine cut-off values for significant predictors, continuous variables were converted into dichotomous categories. The optimal cut-off values were then determined using the likelihood-ratio method, which assessed how well different thresholds fit the model. Statistical analyses were performed using SAS and SPSS software (version 9.4 and 29, respectively).

## Results

Forty-seven patients were enrolled in this study. Details on patient demographics, tumor characteristics, and treatments are provided in **Table 1**. The median follow-up duration was 34.4 months, and the median patient age was 54 years (range: 18–84 years). Males comprised 75% of the cohort, with the majority of patients (47%) diagnosed at stage III. Epstein-Barr virus (EBV)-correlated disease was observed in 77% of cases, and 85% of patients presented with lymph node involvement (N+ disease). RT alone was administered to 6% of patients, while the remaining patients received concomitant chemoradiotherapy. Induction chemotherapy was administered in 55.3% of cases. Only one patient was treated with PT, with the vast majority (98%) receiving IMRT/VMAT. The mean weight loss during treatment was 9% (range: 2.28% to 25.85%). Response to treatment, assessed with PET/CT three months after the end of RT, was known for 44 patients, of which 43 achieved a complete response. Two patients experienced disease recurrence, 6 months and 24 months after treatment. There was one death attributed to NPC. The 2-year and 5-year loco-regional control rates were both 95%, and 2-year and 5-year overall survival rates were 100% and 93%, respectively.

**Table 1:** Patient and treatment characteristics.

Variable	Statistic	All
**Age**	N	47
	Mean	53.38
	Std	14.735
	Median	54.00
	IQR	(44.00; 65.00)
	Range	(18.00; 84.00)
**Gender**		
Female	n/N (%)	12/47 ( 25.53%)
Male	n/N (%)	35/47 ( 74.47%)
**Histology**		
Others	n/N (%)	12/47 ( 25.53%)
Keratinizing SCC	n/N (%)	17/47 ( 36.17%)
Non keratinizing SCC	n/N (%)	18/47 ( 38.30%)
**EBV status**		
0	n/N (%)	11/47 ( 23.40%)
1	n/N (%)	36/47 ( 76.60%)
**T classification**		
1	n/N (%)	14/47 ( 29.79%)
2	n/N (%)	13/47 ( 27.66%)
3	n/N (%)	11/47 ( 23.40%)
4	n/N (%)	9/47 ( 19.15%)
**N classification**		
0	n/N (%)	7/47 ( 14.89%)
1	n/N (%)	22/47 ( 46.81%)
2	n/N (%)	12/47 ( 25.53%)
3	n/N (%)	6/47 ( 12.77%)
**Stage (AJCC 8^th^ edition)**		
1	n/N (%)	1/47 ( 2.13%)
2	n/N (%)	11/47 ( 23.40%)
3	n/N (%)	21/47 ( 44.68%)
4	n/N (%)	14/47 ( 29.79%)
**Bulky N-disease (>15mm)**		
Yes	n/N (%)	42/47 ( 89.36%)
No	n/N (%)	5/47 ( 10.64%)
**Induction chemotherapy**		
Yes	n/N (%)	21/47 ( 44.68%)
No	n/N (%)	26/47 ( 55.32%)
**Concomitant chemotherapy**		
Yes	n/N (%)	44/47 ( 93.62%)
No	n/N (%)	3/47 ( 6.38%)
**Initial weight (pre RT)**	N	45
	Mean	80.81
	Std	14.174
	Median	80.60
	IQR	(71.20; 91.10)
	Range	(54.30; 110.00)
**Initial weight (kg)**		
<90kg	n/N (%)	38/47 ( 80.85%)
>90kg	n/N (%)	9/47 ( 19.15%)
**Weight loss (kg)**	N	47
	Mean	6.73
	Std	3.933
	Median	6.90
	IQR	(3.70; 9.20)
	Range	(-0.30; 16.20)
**% Weight loss**	N	45
	Mean	8.59
	Std	4.057
	Median	8.56
	IQR	(5.77; 11.24)
	Range	(-0.33; 15.40)
**Weight loss**		
<10%	n/N (%)	28/47 ( 59.57%)
>10%	n/N (%)	19/47 ( 40.43%)
**BMI_PRE_RT**	N	43
	Mean	26.54
	Std	4.219
	Median	26.80
	IQR	(23.99; 28.77)
	Range	(16.86; 36.42)
**Response to RT (CR=1; PR=2; PD=0)**		
Persistent disease	n/N (%)	2/47 ( 4.26%)
Complete response	n/N (%)	42/47 ( 89.36%)
Partial response	n/N (%)	3/47 ( 6.38%)
**Status (NED=1; AWD=2; DOC=3; DOD=0)**		
DOD	n/N (%)	1/47 ( 2.13%)
NED	n/N (%)	41/47 ( 87.23%)
AWD	n/N (%)	4/47 ( 8.51%)
DOC	n/N (%)	1/47 ( 2.13%)

SCC, squamous cell carcinoma; BMI, body mass index; RT, radiotherapy; DOD, death of disease; NED, no evidence of disease; AWD, alive with disease; DOC, death of other causes.

**Figure 1:**
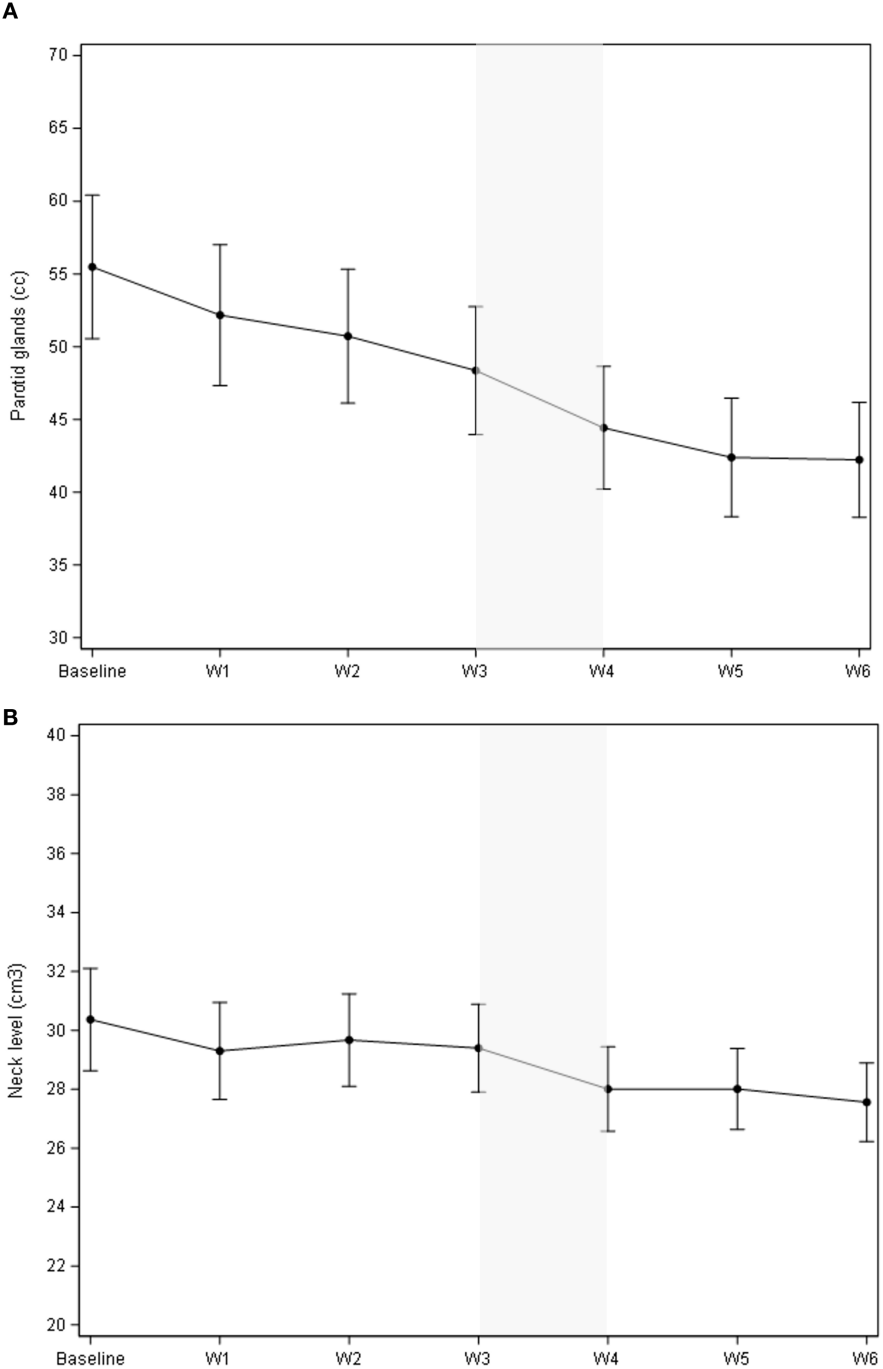
Mean volumetric changes (cc) of **(A)** (mean of both) parotid glands during RT, **(B)** the body contour at the level of cervical vertebra C3 (cm^3^). **(A)**: Mean change of the parotid glands vs. baseline was -11.1 cc (95% CI -13.7; -8.41), p<.0001; mean change vs. the previous week was -3.94 cc (95% CI -6.16; -1.72), p=0.0006. **(B)**: Mean change of the body contour at C3 vs. baseline was -2.36 cm^3^ (95% CI -1.99;-1.78), p<.0001); mean change vs. the previous week was – 1.39 cm^3^ ( 95% CI -1.99;-1.078), p<.0001).

**Figure 2:**
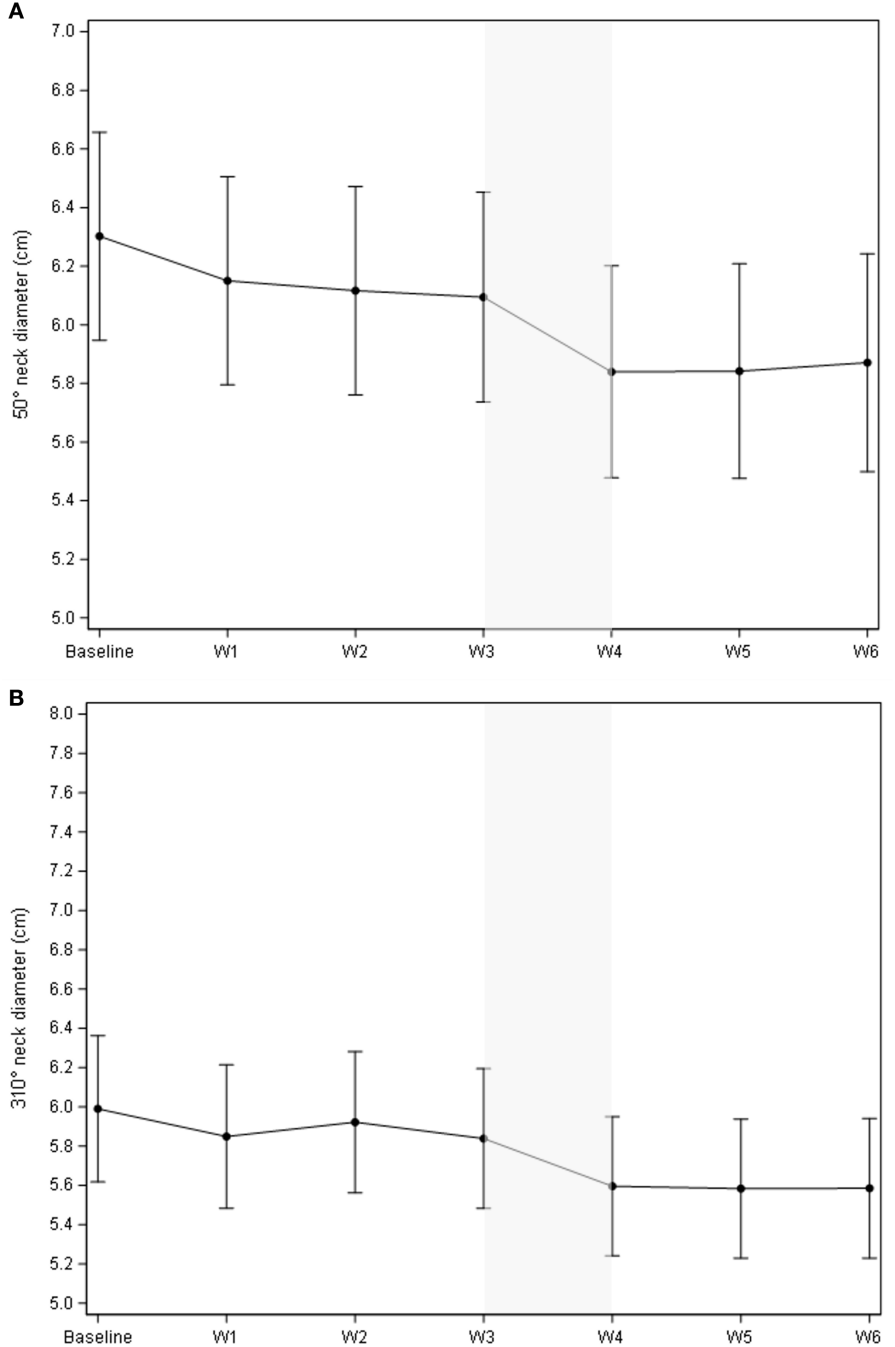
Evolution of the body contour at the 50° **(A)** and 310° **(B)** neck diameter (cm). The most significant decrease in radii occurred between weeks 3 and 4, for both the 50° and 310° measurements, exhibiting the steepest slope coefficient during this period. **(A)**: Mean change of the body contour vs. baseline was -0.46 cm (95% CI -0.63; -0.30), p<.0001; mean change of the body contour vs. the previous week was -0.25 cm (95% CI -0.40; -.011), p=0.0005.

**Figure 3:**
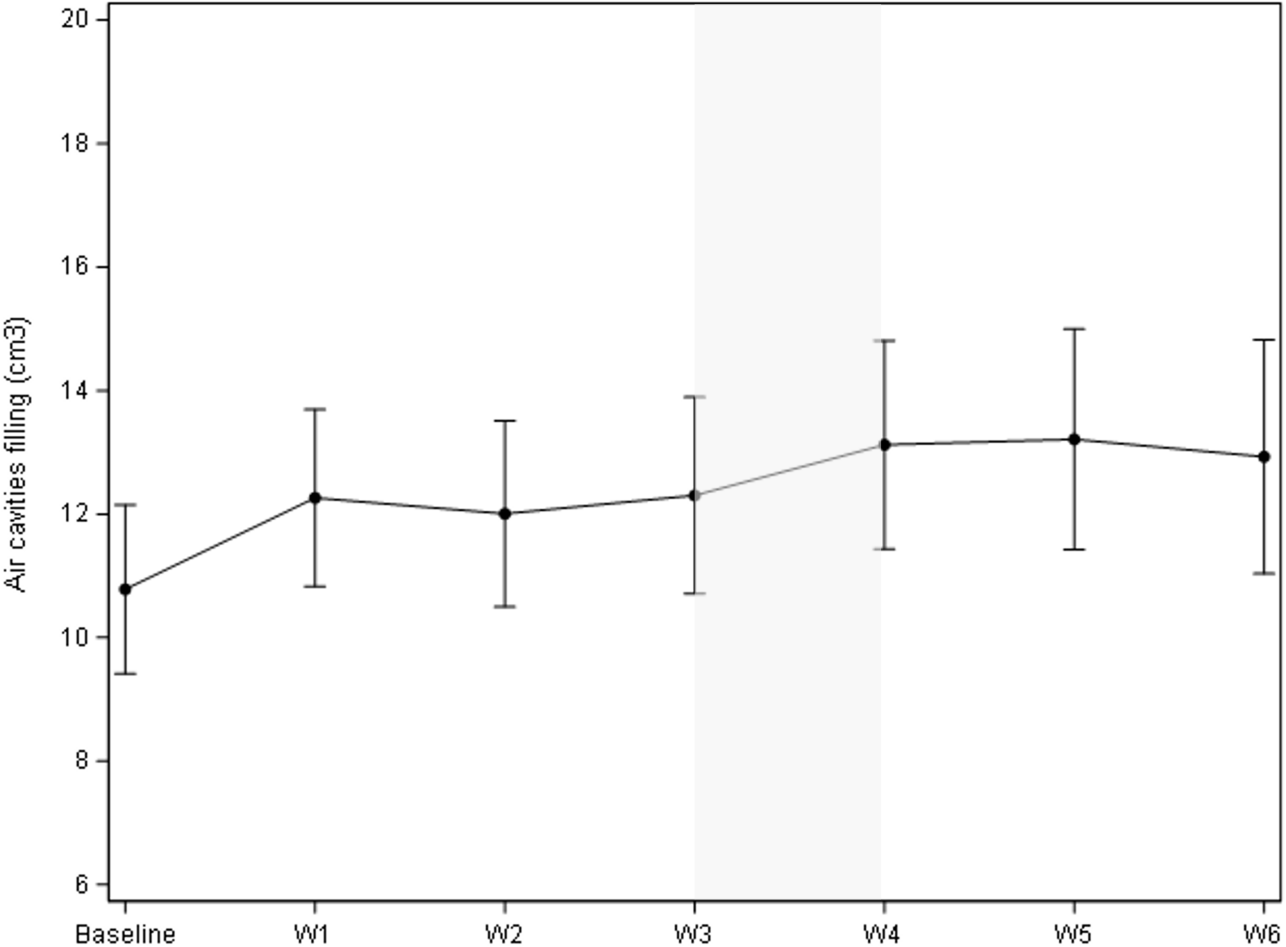
Evolution of air cavity diameter (cm^3^) during treatment. Mean change of the air cavity diameter vs. baseline was 2.34 cm^3^ (95% CI 1.31;3.37), p<.0001; mean change vs. the previous week was 0.81 (95% CI -0.04;1.68), p=0.05.

As illustrated in **Figure 1A**, the cumulative volume of both ipsilateral and contralateral parotid glands decreased from 55.5 cc to 42.2 cc during treatment. The most significant reduction occurred between the third and fourth weeks, with a median volume decrease of 3.94 cc (p = 0.0006). While no significant changes were observed in body contour at the nasopharyngeal level, a 10% reduction was noted at the neck level (C3) (**Figure 1B**). The most significant body contour changes occurred between weeks 3 and 4 of treatment (-1.4 cc; p < 0.001). Additionally, both the 50° and 310° neck diameters showed significant reductions during RT, with the most pronounced decrease occurring between weeks 3 and 4 (**Figure 2**). The air filling of the nasopharyngeal air cavity increased by 2.34 cm³ during the first four weeks of treatment (**Figure 3**) (p = 0.05), while no significant changes were observed in maxillary cavity filling.

**Figure 4:**
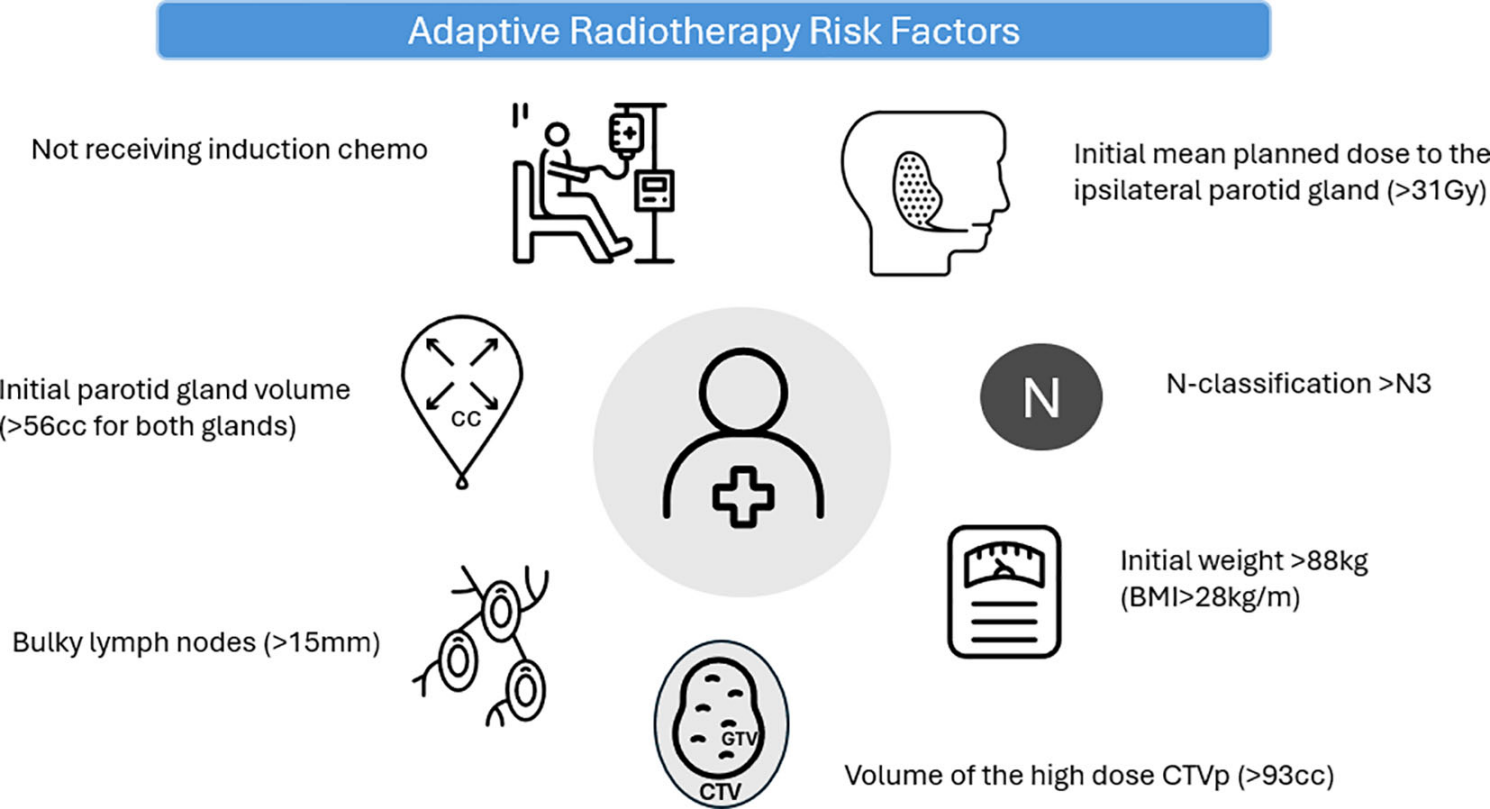
Identified risk factors associated with the need for adaptive radiotherapy in patients with nasopharyngeal carcinoma.

Linear mixed modeling identified bulky disease and a greater initial parotid gland volume as significant predictors for parotid gland volume reduction (HR 9.5, p = 0.005; HR 0.31, p < 0.001, respectively). Significant predictors for body contour change included high N-classification, higher initial weight, bulky lymph node disease, higher initial mean planned dose to the ipsilateral parotid gland, and larger initial CTV-P volume. Patients who received induction chemotherapy exhibited less change in nasopharyngeal air cavity filling compared to those who received only concomitant chemoradiotherapy (HR 2.4; p < 0.01). Cut-off thresholds for large anatomical changes were established, based on descriptive dichotomization, for bulky lymph nodes (> 15mm), initial parotid gland volume (> 56 c cumulative volume for both glands), N-classification (≥ N3), initial weight (> 88kg, corresponding with a BMI higher than 28 kg/m²), initial mean planned dose to the ipsilateral parotid gland (> 31 Gy), and the volume of the high-dose CTV-P (> 93 cc). **Figure 4** illustrates the identified risk factors associated with the need for adaptive radiotherapy in patients with nasopharyngeal carcinoma.

## Discussion

Since ART approach can be initiated both in reaction to a specific event or at predetermined time intervals throughout the course of RT, many questions are rising concerning patient selection, time regimen and dependable methods for triggering re-CT and/or replanning. NPC patients represent an ideal population for studying ART due to their high rate of viral association and favorable response to RT (11). Research by Brown et al. has shown that NPC patients are more likely to require replanning compared to other HNC patients (12). Yang et al. have highlighted that anatomical changes, such as tumor shrinkage, nodal mass reduction, and weight loss, are frequently observed in NPC patients during RT (13). Additionally, Cheng et al. have noted that NPC patients often receive doses to target volumes (TVs) and critical organs at risk (OAR), such as the brainstem and optic structures, that approach or exceed tolerance levels (14). This underscores the critical role of ART in ensuring dose acceptability and optimizing treatment outcomes for these patients.

This study aimed to identify predictive parameters for the need for ART and re-planning in patients with NPC and to establish the optimal timing for replanning. The analysis incorporated potential pre-treatment selection criteria from recent literature (12, 15–20). Parotid gland volumes were monitored for treatment impact related to xerostomia, while air cavities and maxillary sinus volumes were assessed to understand tumor regression and variations affecting proton planning, respectively. Our findings suggest that the third week of the treatment is the most suitable time for replanning, based on volumetric changes in the parotid glands, body contour alterations, and air cavity filling. Several pre-treatment predictors for these volumetric changes were identified: higher initial mean dose on the parotid glands, higher initial weight, higher initial BMI, a larger CTV, higher disease stage, initial small volume of the parotid glands, the administration of concomitant chemotherapy and bulkier lymph node disease.

In an additional exploratory analysis, thresholds for these clinical parameters were established. This analysis enables the identification of NPC patients at the time of diagnosis who may be eligible for adaptive radiotherapy (ART) during their radiation treatment. Based on our results, the patient who should be closely monitored during RT to evaluate the need for ART is characterized by N3 or higher classification, presence of a bulky lymph node larger than 15 millimeters, initial BMI exceeding 28 kg/m², mean planned dose to the ipsilateral parotid glands greater than 31 Gy, cumulative volume of the parotid glands greater than 56 cc, high-dose CTV of the primary tumor greater than 93cc, and receiving RT with no prior induction chemotherapy.

Patients who received induction chemotherapy experienced fewer anatomical changes during their treatment. During the first four weeks of treatment, the air filling of the nasopharyngeal cavity increased by 2.34 cm³ (**Figure 3**, p = 0.05), likely due to tumor regression. This observation helps explain why patients who received induction chemotherapy exhibited less change in air cavity filling compared to those who underwent concomitant chemoradiotherapy. Our findings are consistent with several studies that have identified the mean dose to the parotid gland as a significant predictor of parotid gland volume loss (15, 20–28). However, Brouwer et al. identified a lower cut-off value (22.2 Gy) compared to our analysis (31 Gy).

Other predictors reported in the literature include higher initial mean parotid gland dose, higher initial weight, higher BMI, larger clinical target volume (CTV), advanced T and N stages, smaller initial parotid gland volume, overlap between the parotid gland and target volumes, administration of concomitant chemoradiotherapy, and bulkier disease (12, 16–20). In a study by Brown et al. (12), multivariable analysis demonstrated that NPC patients with N2–3 disease, an initial weight over 100 kg, and larger initial nodal sizes had an 80% or higher likelihood of requiring replanning during treatment. While only two patients in our cohort presented with an initial weight exceeding 100 kg, our analysis similarly identified elevated initial body weight as a predictive factor for significant anatomical changes during treatment, with the relevant threshold set at 88 kg.

The effective integration of ART in the clinical management of HNC necessitates precise timing of the intervention. Currently, there is no consensus on the optimal frequency and timing for replanning in HNC patients (22). Our results align with existing evidence, indicating that the most significant anatomical changes tend to occur between the third and fourth week of the RT course (11). Our findings corroborate the conclusions of Brown et al., who suggested that replanning should be initiated at the start of the third week for patients with NPC and during the fourth week for those with oropharyngeal carcinoma (29).

Strengths of our study include the availability of imaging during RT, the use of multiple methods to assess changes in body contour, the consideration of beam angles for PT, and the exclusive focus on NPC patients. However, our study’s retrospective design is a limitation, as is the limited number of patients and the lack of a defined clinically relevant threshold for volumetric change necessitating replanning. In Brown et al.’s study, the need for replanning was determined by radiation oncologists based on the dose to optic structures and the brachial plexus, while Brouwer et al. set their threshold at a 3 Gy mean dose increase to the parotid gland (12, 20, 21,). However, defining such a threshold is challenging. In Brown et al.’s study, only 5 of 110 patients (4.5%) were selected for replanning, an observation that aligns with our clinical experience (12). Furthermore, we need to consider that the high threshold for initiating ART could be partially attributed to the labor-intensive nature of the ART process. We have attempted to address this issue by converting continuous variables into dichotomous categories for establishing cut-off thresholds that most reliably predict the likelihood of a patient experiencing anatomical changes throughout the treatment. However, this approach knows several limitations. First of all, the analysis was performed on only 47 patients, and the clinical parameters were derived from an initial univariate analysis (linear model testing). Secondly, although statistical significance was calculated during the determination of cut-off values, relying solely on significance testing may present an overly optimistic view of the thresholds’ accuracy. In any case, our thresholds are hypothesis-generating and validation of these pre-treatment clinical predictors on a large, prospective dataset is essential before clinical usage. Furthermore, in a subsequent study, it will be important to evaluate whether the reported volumetric changes lead to dosimetric implications that may affect acute or late toxicity. A suitable approach would be to incorporate the dosimetric effects into existing Normal Tissue Complication Probability (NTCP) modeling for xerostomia, dysphagia, and tube feeding dependence (30).

In the evaluation of the 310° and 50° NPC radii on both the planning CT and subsequent CBCTs, the anterior point of the dens axis serves as a consistent and clearly identifiable landmark on both imaging modalities. This outermost point of the body can be reliably used to establish the angular radius relative to the imaging midline. However, a key limitation is that small variations in patient orientation—such as pitch, roll, or rotation, often in combination—within the immobilization mask and 3D treatment couch can introduce deviations in the measured radii. These inaccuracies may arise from systematic factors (e.g., treatment couch sagging compared to the CT simulation couch) or random day-to-day variations in patient positioning. Despite these potential sources of error, significant and clinically relevant reductions in radii were still observed.

## Conclusion

The time frame between the 15^th^ and 20^th^ fraction is advised as the optimal timing of evaluating the patient’s need for ART. This study identified several clinical criteria that may serve as indicators of significant volume changes in patients undergoing RT for NPC: N3 or higher classification, presence of a bulky lymph node larger than 15 millimeters, initial BMI exceeding 28 kg/m², mean planned dose to the ipsilateral parotid glands greater than 31 Gy, cumulative volume of the parotid glands greater than 56 cc, high-dose CTV of the primary tumor greater than 93 cc, and receiving RT with no prior induction chemotherapy. To further clarify confounding factors, it will be essential to repeat multivariable analyses and establish cut-off values in larger, well-selected patient cohorts.

## Data Availability

The original contributions presented in the study are included in the article/**Supplementary Material**. Further inquiries can be directed to the corresponding authors.
