# Supplementary material for: Strategy to select nasopharyngeal cancer patients for adaptive radiotherapy
Source: Front Oncol. 2025 Sep 29;15:1653060. doi: 10.3389/fonc.2025.1653060 (PMC12515621; doi:10.3389/fonc.2025.1653060)
Supplement: Supplementary file 1 [file DataSheet1.docx]

Supplementary Materials


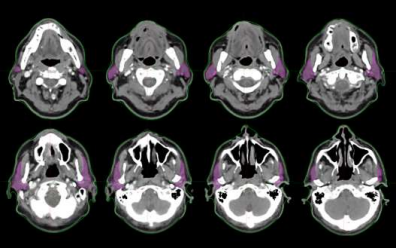
*Figure 1A. Parotid glands contouring*

*
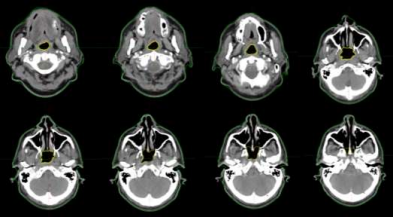
Figure 2A. Air cavities contouring*

*
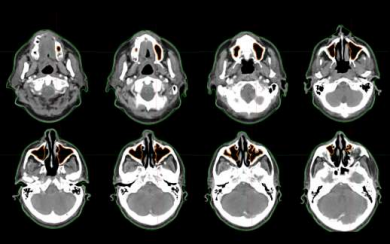
Figure 3A. Maxillary sinuses contouring*

*
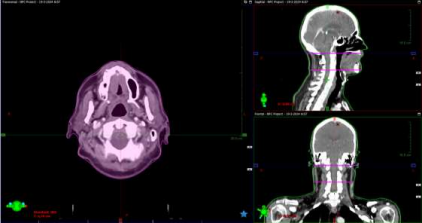
Figure 4A. Nasopharyngeal Level*

*
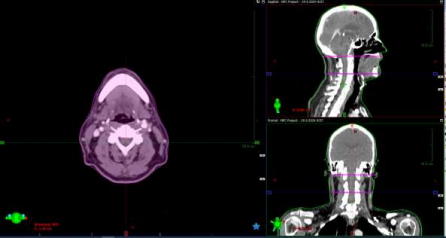
Figure 5A. Neck Level*

*
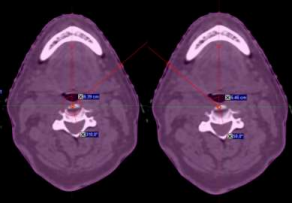
Figure 6A. Nasopharyngeal Level radius angle 50° and 310°°*

*
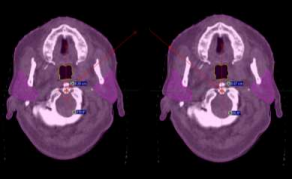
Figure 7A. Neck Level radius angle: 50°- 310°*
